# The Influence of Cold-Working Deformation on the Measurement Accuracy and Stability of Type-K Sheathed Thermocouple Sensors

**DOI:** 10.3390/s26134288

**Published:** 2026-07-06

**Authors:** Jie Chen, Xiaodong Peng, Min Liu, Zheng Sun, Anzhong Zhao, Jixiang Xie

**Affiliations:** 1 School of Intelligent Manufacturing and Automobile, Chongqing Polytechnic University of Electronic Technology, Chongqing 401331, China; sz04021993@163.com (Z.S.); jixiangxie@stu.cqu.edu.cn (J.X.); 2 Department of Material Science and Engineering, Chongqing University, Chongqing 400044, China; pxd@cqu.edu.cn; 3 Chongqing Material Research Institute Co., Ltd., Chongqing 400707, China; ccliumin@139.com (M.L.); ncfma06@126.com (A.Z.)

**Keywords:** thermocouple, electromotive force (EMF) drift, cold-working, stability

## Abstract

**Highlights:**

**What are the main findings?**

- Cold working enhances negative EMF drift and hysteresis, dominated by the KP alloy.
- Higher deformation induces larger EMF drift during long-term aging at 350 °C.
- Aging below 600 °C stabilizes ordered structures and eliminates EMF hysteresis.
- The EMF drift resulting from >28% deformation cannot be recovered even after aging at 700 °C.

**Abstract:**

This study investigates the influence of cold-working deformation on the electromotive force (EMF) calibration characteristics, hysteresis behavior, and long-term stability of the Type-K mineral-insulated metal-sheathed (MIMS) thermocouples used in Combination Fixed In-Core Detector Assemblies for pressurized water reactor nuclear power plants. Reduction ratios of 12%, 28%, and 38% were investigated, and samples were subjected to heating–cooling calibration and in situ aging tests. The results show that increased cold-working deformation leads to greater negative EMF deviation and larger heating–cooling hysteresis, mainly affected by the degradation of the positive KP thermoelement. Cold-working lowers the atomic diffusion activation energy and accelerates element migration, resulting in pronounced EMF drift during isothermal aging at 350 °C for 720 h. After aging below the order–disorder transition temperature, stable ordered structures form in the thermoelement alloys and hysteresis is significantly reduced. However, within the range investigated in this study, deformation above 28% imparts irreversible effects. The EMFs of 28% and 38% deformed samples remained lower than that of the undeformed state even after isothermal aging at 700 °C for 500 h. These findings reveal that excessive cold-working deformation severely impairs the measurement accuracy and long-term stability of the thermocouples, highlighting the necessity of the strict control of drawing deformation to ensure the reliability of nuclear-grade thermocouples under both normal and abnormal reactor operating conditions.

## 1. Introduction

Thermocouples are extensively applied for temperature measurement across advanced industries due to their wide measurement range, high reliability and favorable interchangeability [1,2,3]. Precise monitoring and control of in-core temperatures are fundamental to the safe operation of nuclear reactors [4,5]; this imposes stringent requirements on the long-term stability of temperature sensors. As the primary sensing solution for harsh in-core environments, mineral-insulated metal-sheathed (MIMS) thermocouple temperature sensors are widely used in nuclear reactors, motivating extensive worldwide research into their drift mechanisms and accuracy degradation under harsh nuclear environments [6,7,8,9,10,11].

Upon exposure to fast neutrons and following aging at 565 °C, the electromotive force (EMF) drift of Type-K thermocouples is negative upon heating during calibration and reverses to positive during cooling. This behavior has been attributed to recrystallization of the thermocouple alloys during thermal cycling [9]. After γ irradiation, the positive thermoelement undergoes short-range ordered structural transformation, altering the alloy’s specific heat capacity, reducing the hysteresis loop during heating–cooling calibration and introducing positive reversible drift [12]. In gas-cooled high-temperature reactors, after exposure to 1100–1150 °C for 1500 h, with helium as the cooling gas, thermocouple drifts are around −10 to −50 °C, attributed to Cr diffusion from the positive (KP) to negative (KN) thermoelement, which results in changes in the chemical composition of the thermocouple alloys [6].

The potential drift of a thermocouple temperature sensor is influenced not only by irradiation but also by the chemical composition and physical state of the alloy [13,14,15]. The sheath alloys in MIMS thermocouples might prevent contamination from the service environment and enhance service life, and when Hastelloy X and AISI310 alloys are used as sheath alloys, Mn diffuses into the KN wire and increases the negative drift significantly [16].

When using 316L as the sheath alloy, the EMF drifts at high temperature are caused by inhomogeneous oxidation of the KN wires. Elements such as Cr, Si, and Al diffuse toward the surface, thus reducing their concentrations in the unoxidized area, leading to a sharp decline in the EMF. Alternatively, when Inconel 600 is employed as the sheath alloy, the drift is attributed to the precipitation of Cr and Fe in the grain boundaries or the inner grain, leading to an inhomogeneous composition in the thermocouple wire [17]. M. Scervini et al. [18] devised a double metal-sheathed thermocouple using AISI 310 as the outer layer to suppress oxidation and Ni 270 as the inner layer to block elemental migration from the sheath into the thermoelements.

**Figure 1:**
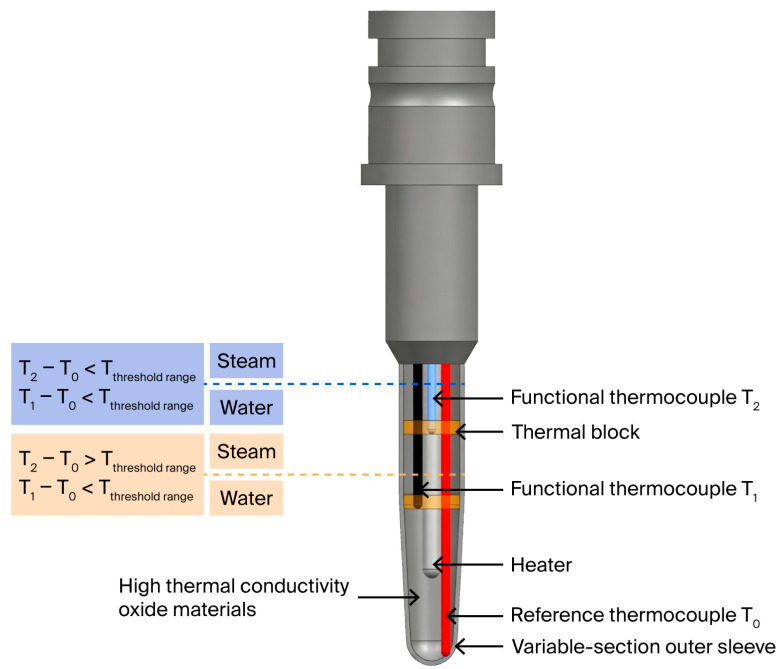
Schematic of the measurement principle of Combination Fixed In-Core Detector Assemblies.

In third-generation commercial pressurized water reactor nuclear power technologies, including Hualong One and CAP1400, a critical improvement is the integration of temperature sensors and water-level sensors combination fixed in-Core detector assemblies. In these assemblies, fixed-point monitoring of reactor pressure vessel water level and temperature monitoring at the reactor core outlet are realized simultaneously. The integrated in-core detector assemblies are constructed by integrating sheathed heaters and thermocouple sensors (Figure 1). Measurements are taken based on the remarkable difference in the heaters’ heat transfer coefficients between gaseous/vapor and liquid media, thus converting water-level monitoring into a temperature difference measurement. The temperature difference between the heated functional thermocouple sensor and the unheated reference thermocouple sensor (which is always immersed in liquid water) is compared against a preset threshold. When the temperature difference remains within the threshold range, the functional thermocouple is submerged in water, indicating that the water level is higher than the installation position of the functional thermocouple. Once the temperature difference exceeds the threshold, the heater exhibits deteriorated heat dissipation performance, implying that the functional thermocouple is exposed to a steam atmosphere and the water-level has dropped below the detection elevation, fulfilling the requirement for fixed-point water level monitoring inside the nuclear reactor. In addition, the unheated reference thermocouple inside the detector is placed at the reactor core outlet, allowing the detector assembly to measure the core outlet temperature.

Improving the sensitivity of gas–liquid interface detection is a key technical challenge when manufacturing combination fixed in-core detector assemblies. During the assembly process, the thermocouple, heater, thermal block, high-thermal-conductivity oxide materials and outer sleeve of the detector assembly are assembled first, followed by re-drawing and diameter reduction to achieve a tight fit among the three components. This process enhances the heat transfer efficiency at the heater’s surface, enabling the thermocouple to quickly sense temperature changes and thereby improving the sensitivity of fixed-point water-level monitoring and detector assembly warnings.

However, after re-drawing and diameter reduction of the thermocouple during assembly integration, cold-working stress will be generated, and heat treatment cannot be performed to eliminate this. This may lead to changes in the thermocouple’s thermoelectric characteristics. The unheated reference thermocouple inside the detector, which functions as a core outlet temperature monitor to evaluate the heat removal status of the reactor core coolant, is classified as a nuclear safety-level sensor that must be present in nuclear power monitoring equipment. It has high technical requirements in terms of measurement accuracy, long-term stability and reliability.

Up to now, few studies have focused on the quantitative influence of assembly-induced cold-working deformation on the EMF characteristics, hysteresis behavior and long-term stability of nuclear-grade MIMS Type-K thermocouples. The evolution of the thermoelectric properties under normal and abnormal reactor temperatures remains unclear. Therefore, in this study, systematic experiments are carried out to reveal the effects of drawing deformation (12%, 28%, 38%) on calibration characteristics, heating–cooling hysteresis and high-temperature aging stability. The results can provide key data support and a theoretical basis for the deformation control, reliability design and safety assessment of in-core detector assemblies.

The conclusions are of great engineering value regarding improving the measurement accuracy and service stability of nuclear-grade thermocouples, and are also important for further ensuring the safe and stable operation of pressurized water reactor nuclear power plants.

## 2. Materials and Methods

Type-K NiCr-NiAl thermocouple wires (with the calibration characteristics shown in Table 1, Isabellenhütte Heusler GmbH & Co. KG, Dillenburg, Germany) were assembled with high-purity MgO powder (Lianyungang Fenghai magnesium Industry Co., Ltd., Lianyungang, China) and sheathed 316L stainless steel (Chongqing Material Research Institute Co., Ltd., Chongqing, China) (chemical compositions shown in Table 2, Table 3 and Table 4).

After multiple drawing and annealing processes, the MIMS thermocouple cables were fabricated with an outer diameter of Φ3.2 mm (deformation degree of 0%, i.e., the initial undeformed state). To replicate the effects of assembly stress on the thermocouple sensors, the diameter was further altered to Φ3.0 mm (deformation degree of 12%), Φ2.7 mm (deformation degree of 28%), and Φ2.5 mm (deformation degree of 38%). Cold-working deformation was applied without subsequent heat treatment, so as to simulate the cold-working stress generated during the fabrication of combination fixed in-core detector assemblies.

Cold-working deformation degree % can be calculated by the following formula:

(1) $$\eta =\frac{D_{0}^{2}-D_{1}^{2}}{D_{0}^{2}}\times 100\%$$

where *η* is the cold-working deformation degree (%), $D_{0}$ is the initial outer diameter of the MIMS thermocouple cable before cold-working (mm), and $D_{1}$ is the outer diameter after cold-working (mm).

The dimensions of the thermocouples are illustrated in Table 5.

The hot junction was welded to the sheath as an insulator. The thermocouples were baked at 120 °C for 5 h to remove residual water vapor from the MgO powder. Then, the cold junction of the thermocouple was sealed using epoxy to prevent moisture and air intrusion. The insulation resistance of the thermocouples was measured with a Model ZC-7 analog insulation resistance meter at a DC voltage of 500 V. The minimum insulation resistance at room temperature was greater than 1000 MΩ, and the accuracy class of the instrument was 10.

All performance tests were conducted with duplicate specimen sets to ensure data reliability. Within each set, one specimen was adopted for basic calibration tests, another for single thermoelement versus platinum calibration, and a third for heating and cooling calibration tests. For the high-temperature aging tests, one specimen was used for aging at 350 °C, while three specimens were applied separately for aging at 500 °C, 600 °C and 700 °C. After aging, all specimens were subjected to repeated heating and cooling calibration tests.

These calibration tests were performed to evaluate the reliability of the MIMS thermocouples in accordance with ITS-90 (The International Temperature Scale of 1990) in a standard laboratory. The thermal EMF–temperature characteristics of the thermocouples were measured at 100 °C and 200 °C in a CS-604 oil bath, with calibration against a first-class standard platinum resistance thermometer (SPRT).

For temperatures above 300 °C, thermal EMFs were measured using a GL-4 tube furnace (Shanghai Jialing Electronic Technology Co., Ltd., Shanghai, China) and calibrated against a first-class standard type-S thermocouple. The tube furnace featured a uniform temperature zone whose center deviated from the geometric center along the axis by no more than 10 mm. Within this zone, which extended at least 60 mm in length and had a radius of 14 mm, the maximum temperature variation did not exceed 1 °C.

The calibration tests were conducted at a metrology institute in compliance with the requirements of national metrological verification regulations. During heating calibration, the heating rate of the tube furnace was controlled at 5–10 °C/min, and the furnace cooling rate was adopted in the cooling stage. At each calibration temperature point, the EMF was measured four times and the average value was taken. The expanded uncertainty (U) was estimated to range from approximately 0.05 °C to 0.15 °C (coverage factor k = 2), corresponding to a thermoelectric voltage interval of roughly 2 µV to 6 µV.

Long-term in situ aging tests were carried out at 350 °C, 500 °C, 600 °C, and 700 °C in a GL-4 tube furnace with an immersion depth of 310 mm for each sample. The hot junction of the tested thermocouple and a standard type-S thermocouple was bundled together and fastened using two or three Nichrome wires; thus, all hot junctions were located in an identical temperature field. A working standard type-S thermocouple was adopted as the reference to measure the actual calibration temperature. The cold junctions of the thermocouples were placed in an ice-point thermostat with a reserved wire length of about 220 mm. Data recording was started once the furnace temperature stabilized within ±2 °C of the target calibration point, with a temperature variation rate no higher than 0.1 °C/min.

The measured thermoelectric EMF at this moment was defined as the initial measurement value $E_{0}$, while the corresponding time was recorded as the start time of the stability test. The furnace was kept under continuous isothermal conditions, and thermoelectric EMF values were recorded at regular hourly intervals. EMF stability is defined as the maximum deviation ${∆E=E-E}_{0}$ between the measured EMF E and the initial EMF $E_{0}$. This parameter characterizes the stability of thermocouples.

## 3. Results and Discussion

### 3.1. Calibration Characteristics

#### 3.1.1. Basic Calibration Characteristics

**Figure 2:**
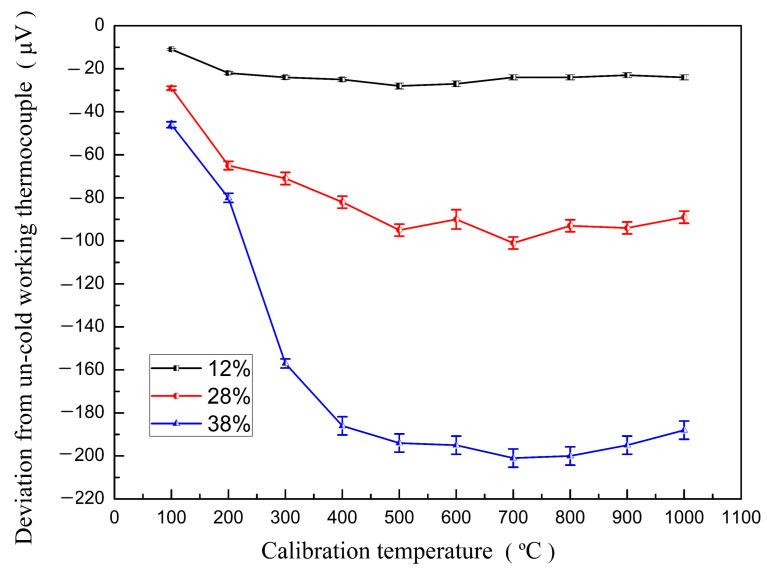
EMF deviations of cold-worked sheathed thermocouples relative to the undeformed reference.

Figure 2 shows the deviations in EMF of the cold-worked sheathed thermocouples relative to the undeformed reference over the temperature range of 100 °C to 1000 °C. Compared with the undeformed sample, all cold-worked samples exhibited negative EMF deviations, and the magnitude of this deviation generally increased with the deformation degree. In the low-temperature range of 100 °C to 500 °C, the negative EMF deviation increased rapidly with rising temperature, showing a steep downward trend. At 100 °C, the EMF decreased by approximately 23 μV in the sample with 12% deformation, 29 μV in the 28% deformed sample, and 42 μV in the 38% deformed sample. In the calibration temperature range of 500 °C to 1000 °C, the EMF drift tended to stabilize: for the samples with 28% and 38% deformation, the EMF drift in the high-temperature region stabilized at approximately −194 μV and −94 μV, respectively. These results are consistent with the findings of Zhao Rong-bin et al. [19], indicating that type-K MIMS thermocouples exhibit negative EMF drift after cold-working deformation.

**Figure 3:**
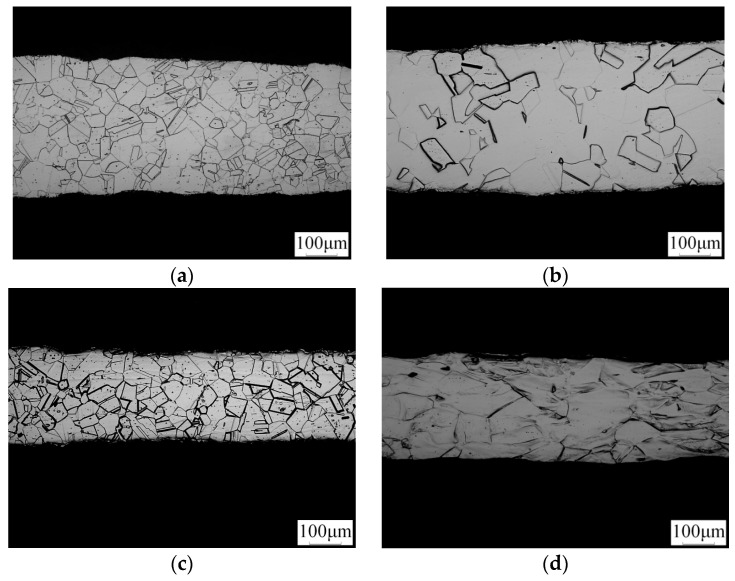
Microstructures of MIMS thermocouples under different cold-working deformations: (**a**) undeformed KP alloy; (**b**) undeformed KN alloy; (**c**) KP alloy at 38% deformation; (**d**) KN alloy at 38% deformation.

To further investigate the effect of cold-working on EMF drift, metallurgical analysis was performed on the cross-sections of the thermocouples. As depicted in Figure 3, the microstructures of positive and negative thermocouple wires, sectioned along the drawing direction, were observed, corresponding to deformation degrees of 0% and 38%, respectively. For the sample without cold-working (0% deformation), distinct grain boundaries were observed in both the positive and negative thermocouple wires. After 38% cold-working deformation, the microstructure of the positive KP alloy was significantly refined, exhibiting fine recrystallized grains. For the negative KN alloy, a typical dilute solute alloy, the grain boundaries underwent severe distortion and fragmentation under drawing stress. The original intact grain structure was disrupted, and a fibrous deformed microstructure was formed. This finding indicates that cold-working exerts differential effects on the microstructures of positive and negative thermoelement alloys.

The aforementioned macroscopic EMF variations exhibit a clear intrinsic correlation with the microstructural evolution. The direct experimental evidence in this study, obtained through metallographic observations, confirmed that cold-working deformation induced grain boundary fragmentation, grain refinement and microstructure distortion, corresponding to an increased density of crystal defects within the thermoelements as the deformation degree increased. Meanwhile, the calibration results showed that the magnitude of negative EMF deviation also rose significantly with increasing deformation. This demonstrates a consistent trend between microstructural changes and macroscopic property variations.

According to classical electron scattering theory, drawing stress distorts the crystal lattice, intensifies electron scattering, modifies the Brillouin zone of crystals, changes the electron density of alloys, raises the Fermi energy and thereby causes changes in electron transport behavior [19,20]. It can be inferred from the experimental observations and classical theory that the abundant crystal defects introduced through cold-working deformation alters the electron transport properties, which is the main cause of the negative EMF drift. A higher cold-working deformation degree results in more defects in the thermoelement alloy, more pronounced changes in electron transport behavior and accordingly a greater magnitude of negative EMF drift.

#### 3.1.2. Calibration Characteristics of a Single Thermoelement vs. Platinum

The hot end of the sheathed thermocouple was fabricated into a welded junction, where the positive thermoelement, negative thermoelement and pure platinum wire were welded together. Measuring the single-element EMF relative to platinum enables a quantitative evaluation of the individual effects of the cold-working deformation degree on positive and negative thermoelements. Table 6 summarizes the variations in single-element EMF compared to platinum for the thermocouple with a 28% deformation degree from 100 °C to 1000 °C. After cold-working deformation, the EMF drift of the KN thermoelement was markedly smaller than that of the KP thermoelement. Accordingly, the overall stability of the thermocouple’s EMF depends primarily on the positive KP thermoelement. This conclusion is consistent with the findings reported by Pollock et al. [21]. Nevertheless, slight differences in potential drift curves can be observed, which originate from different cold-working deformation modes and deformation degrees [22].

At 28% deformation, the KP thermoelement exhibited a far larger EMF reduction from 100 °C to 400 °C than its KN counterpart. In the high-temperature range of 500–1000 °C, KP’s negative EMF drift ranged from −122 μV to −132 μV, substantially exceeding that of KN (−23 μV to −30 μV) in the same interval. Clearly, KP’s negative EMF drift is significantly larger than that of KN.

This indicates that the overall potential stability of the thermocouple is dominated by the positive KP thermoelement. The overall calibration characteristic of the thermocouple is determined by the coupled response of its positive and negative thermoelements, as defined by Equation (2) fined by Equation (2):

(2) $$\Delta EMF_{Total}=\Delta EMF_{\mathrm{KP}}-\Delta EMF_{KN}$$

After cold-working deformation, the negative drift of the KN thermoelement partially compensates for the drift of the KP thermoelement. As a result, the overall calibration curve of the thermocouple strongly matches the KP-versus-platinum characteristic. In the 100–400 °C range, EMF exhibited a sharp negative decline at 38% deformation, while the decay rate slowed remarkably above 500 °C.

The fabrication process for sheathed thermocouple cables is summarized as follows: post-drawing samples were heat-treated at 900–1070 °C for ~20 min, followed by air cooling to room temperature. During thermoelement–platinum calibration tests, samples were held at 900 °C and 1000 °C for 10–20 min for temperature stabilization. However, using this heat treatment method failed to recover the original EMF performance of cold-worked thermocouples. The discrepancy between calibration heat treatment and conventional post-processing heat treatment results in distinct metallurgical states in the thermoelement alloys.

#### 3.1.3. EMF Characteristics During Heating and Cooling Processes

**Figure 4:**
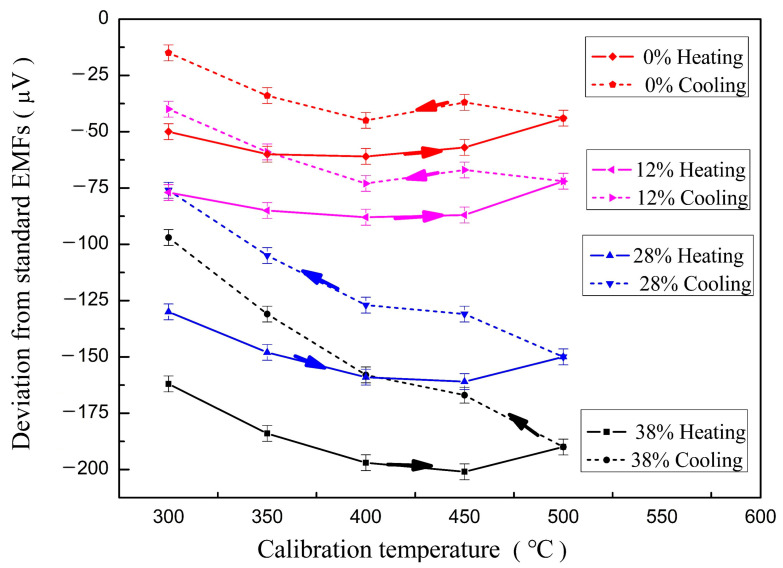
Deviation in EMF from standard values for cold-worked thermocouples during heating–cooling calibration. The arrows indicate the direction of temperature change in the heating–cooling calibration cycle.

A prominent characteristic of Type-K thermocouples is the occurrence of thermoelectric EMF hysteresis between heating and cooling calibrations in the temperature range of 200 °C to 600 °C [23]. To comprehensively assess the impact of cold-working deformation on the performance of the MIMS thermocouple sensor, a heating–cooling calibration test was performed between 300 °C and 500 °C (Figure 4).

In the absence of cold-working deformation, the maximum EMF hysteresis loop during heating and cooling was 35 μV at 300 °C. When the sample was subjected to 12% cold-working deformation, it exhibited almost the same hysteresis curves as the undeformed sample, with a hysteresis difference of only approximately 1 μV at 300 °C and 400 °C. The EMF hysteresis difference increased gradually with increasing deformation degree: when the deformation degree reached 38%, the maximum EMF hysteresis difference was observed at 300 °C, reaching 65 μV. However, as the calibration temperature increased, the EMF hysteresis difference decreased gradually to 34 μV.

The newly manufactured sheathed thermocouple underwent a final annealing process at 1070 °C, followed by air cooling to room temperature, which resulted in a disordered crystal structure. Therefore, the EMF was relatively low during heating calibration. During the cooling calibration, an order–disorder transition occurred, which induced an EMF hysteresis loop. This order–disorder transition is an atomic diffusion process. Cold-working introduces vacancies and dislocations into the alloy, enhancing lattice instability. The increased internal energy of the deformed alloy reduces the diffusion activation energy, and both thermal equilibrium and non-equilibrium vacancies participate in atomic diffusion, thereby accelerating atomic migration [24]. More ordered structures were formed in the sheathed thermocouple with a deformation degree of 38% during cooling, leading to the maximum EMF hysteresis discrepancy.

### 3.2. EMF Stability

#### 3.2.1. In Situ Aging for 720 h at 350 °C

Type-K thermocouple sensors are mainly used for core outlet temperature measurements in large pressurized water reactors (PWRs), where normal operating temperatures are around 330 °C to 340 °C. “Hualong-1” nuclear power technology does not specify requirements for the long-term EMF stability of thermocouple sensors under normal operating temperatures. In contrast, CAP1400 nuclear power technology stipulates that the potential drift of sheathed thermocouple sensors for core outlet temperature measurement should not exceed ±0.3 °C (equivalent to approximately ±12 μV for Type-K thermocouples) after aging at 350 °C for 720 h. For the 300 MW Chashma C3/C4 nuclear power units in Pakistan, technical requirements specify that the EMF drift under the same conditions should be no more than ±0.2 °C (equivalent to approximately ±8 μV).

Temperature is calculated via the equation $T=\frac{EMF}{S}$, where S is the Seebeck coefficient of the Type-K thermocouple. In this study, experimental investigations were conducted to evaluate the above technical specifications.

**Figure 5:**
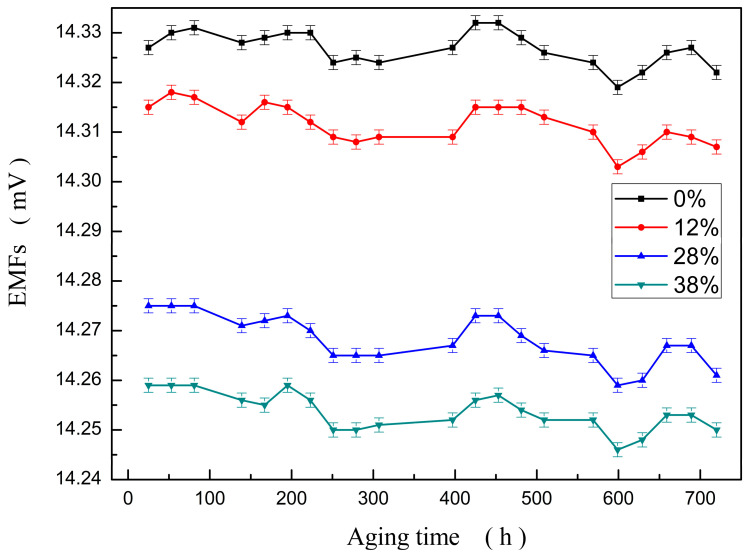
EMF stability of sheathed thermocouples at 350 °C after cold-working deformation.

Figure 5 illustrates the EMF variation of the sheathed thermocouples during aging at 350 °C for 720 h. Relative to the initial calibration EMF, ${∆E=E-E}_{0}$, the EMF drifts after cold-working deformation were −8 μV (0% deformation), −12 μV (12% deformation), −16 μV (28% deformation) and −13 μV (38% deformation), respectively. The maximum EMF fluctuation ($E_{MAX}-E_{MIN})$ reached 13 μV (0% deformation), 15 μV (12% deformation), 16 μV (28% deformation) and 13 μV (38% deformation), respectively.

Comparing the measured drift values with the technical specifications, the undeformed (0%) sample satisfies both the CAP1400 (±0.3 °C/±12 μV) and Chashma unit (±0.2 °C/±8 μV) requirements, while the 12% deformed sample meets the CAP1400 limit but marginally exceeds the Chashma specification. In contrast, both the 28% and 38% deformed samples exceed the drift limits of both standards, indicating that excessive cold-working deformation compromises the long-term stability of the thermocouple, which may not meet the stringent reliability requirements for core outlet temperature measurement in nuclear reactors.

The diffusion activation energy of the thermoelement alloys is relatively low after cold-working. During aging at 350 °C for 720 h, atomic diffusion proceeds more rapidly in thermocouples with higher cold-working deformation degrees, leading to more significant thermoelectric EMF fluctuations.

Although the 38% deformed sample showed a slightly lower drift value (−13 μV) than the 28% deformed sample, this value was still higher than that of the undeformed sample. This may be due to the higher degree of work hardening, local microstress relaxation, or recovery effects induced by heavy deformation. The trend in this phenomenon is not fully monotonic as theoretically expected, and the underlying mechanism should be further confirmed by systematic microstructural characterizations.

#### 3.2.2. Aging for 500 h at 500 °C to 700 °C

**Figure 6:**
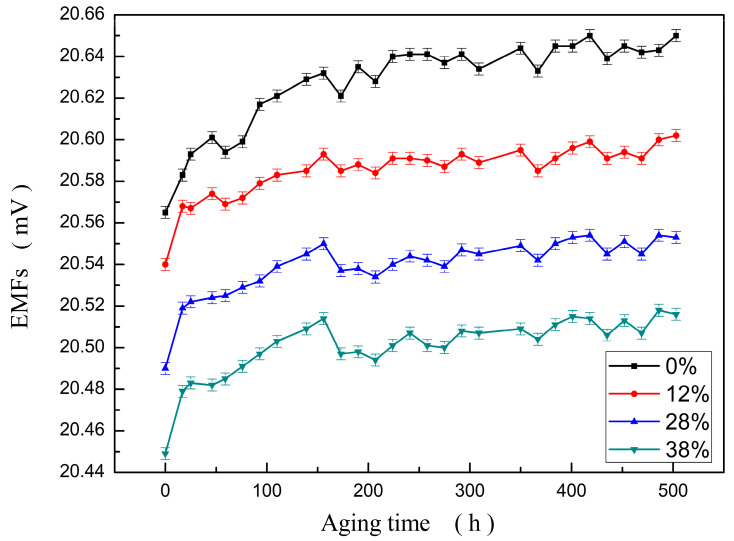
EMF stability of sheathed thermocouples at 500 °C after cold-working deformation.

**Figure 7:**
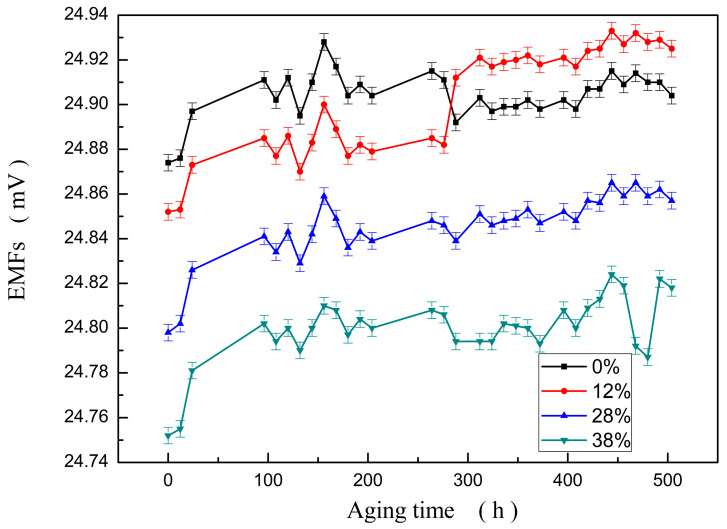
EMF stability of sheathed thermocouples at 600 °C after cold-working deformation.

**Figure 8:**
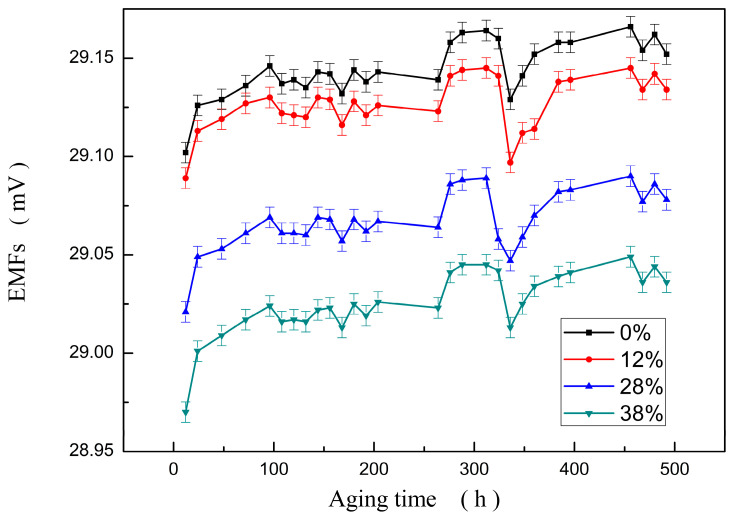
EMF stability of sheathed thermocouples at 700 °C after cold-working deformation.

During the manufacture of combination fixed in-core detector assemblies with sheathed thermocouples, the thermocouples are integrated with heating elements. In particular, functional thermocouple sensors are arranged in close proximity to the heating elements; thus, their service temperature exceeds the normal operating temperature of the reactor core. Meanwhile, reference thermocouples also serve to monitor the core outlet temperature. In cases of abnormal reactor coolant heat removal or power imbalance, the core outlet temperature will increase significantly. Accordingly, the EMF stability of thermocouples under abnormal reactor operating conditions should be considered in addition to that under normal operating conditions. A comparative study was performed to investigate the effects of cold-working on the EMF stability of sheathed thermocouples after in situ aging at 500 °C, 600 °C and 700 °C for 500 h (Figure 6, Figure 7 and Figure 8).

During aging at 500 °C (Figure 6), the maximum EMF fluctuations ($E_{MAX}-E_{MIN}$) of the thermocouples were 85 μV (0% deformation), 62 μV (12% deformation), 64 μV (28% deformation) and 69 μV (38% deformation). EMF fluctuation was slightly suppressed in cold-worked samples compared with the undeformed sample.

The undeformed sample (0% deformation) exhibited an initial EMF (E_0_) of 20,565 μV at the initial stage of aging. Its EMF increased continuously and significantly with prolonged aging time, reaching a maximum value at 500 h. The sample with 12% deformation exhibited an overall upward trend in EMF; after approximately 17 h of aging, its EMF recovered to the level of the undeformed state (20,565 μV) and thereafter followed a trend nearly identical to that of the 0% sample. For samples with 28% and 38% deformation, the EMF values were overall lower than those of the 0% and 12% samples and increased at a slower rate. After 500 h of aging, their EMF values remained significantly below the pre-aging initial value (20,565 μV), with the 38% deformed sample showing a slightly lower EMF than the 28% deformed sample. This finding indicates that aging at 500 °C cannot fully reverse the EMF deviation induced by cold-working when the deformation degree exceeds 28%.

Differences were observed in the aging behavior at 600 °C and 700 °C compared with that at 500 °C (Figure 7 and Figure 8). For the thermocouples with 12% deformation, the EMF recovered to the pre-aging level after 24 h of aging at both 600 °C and 700 °C. When the aging temperature exceeds the order–disorder transition temperature of the KP thermoelement alloy [25], the contribution of structural ordering to EMF drift weakens and the drift is mainly attributed to oxidation. For sheathed thermocouples with 28% and 38% deformation, EMF values remained significantly lower than the initial value of the undeformed thermocouple, even after aging at 700 °C for 500 h. Table 7 presents the EMF recovery behavior of the samples before and after aging.

This demonstrates that high-temperature aging at 700 °C for 500 h cannot completely eliminate the EMF deviation induced by cold-working when the deformation degree exceeds 28%. The persistent EMF deviation and increased fluctuation may lead to inaccurate core temperature monitoring, which is a critical consideration for reactor core safety.

As we were limited by the set deformation gradients in this work, the precise critical deformation degree for this irreversible effect could not be determined. It can be further clarified by introducing samples with finer deformation gradients in subsequent studies.

#### 3.2.3. Heating–Cooling Calibration EMF Characteristics After Aging

After aging at 500 °C for 500 h, the maximum difference between the samples’ heating and cooling calibration EMFs was only −6 μV (as shown in Table 8). According to a study by T.G. Kollie et al., the order–disorder transition in sheathed thermocouples may be completed after only 1 h of aging at 500 °C [25]. In conventional calibration processes, the order–disorder transition usually results in a higher cooling calibration EMF compared to the heating calibration EMF. However, after aging at 500 °C and 600 °C for 500 h, the cooling EMFs of all samples were nearly identical to the heating EMFs, and the hysteresis phenomenon almost disappeared (Table 8 and Table 9). These results indicate that stable and optimally ordered structures had formed in the thermoelement alloys during aging, and cold-working deformation exerted no significant influence on the heating–cooling calibration characteristics after aging at 500 °C and 600 °C. From an engineering perspective, this means that even if the thermocouples undergo a certain degree of cold-working deformation during the manufacturing of integrated in-core detector assemblies, they consistently exhibit extremely low hysteresis errors during long-term service at or near the normal reactor operating temperature (330–340 °C), meeting the accuracy requirements for core outlet temperature monitoring.

After aging at 700 °C, the hysteresis difference in the heating–cooling calibration EMF for cold-worked thermocouples became clearer and increased with the deformation degree (as shown in Table 10). This trend is basically consistent with the results illustrated in Figure 4; that is, the EMF hysteresis difference increases as the cold-working deformation degree rises. Since 700 °C is above the inherent order–disorder transition temperature of the KP alloy, Cr atoms continuously diffuse toward the thermoelement surface and form a dense Cr_2_O_3_ oxide layer.

The diffusion of Cr atoms and the oxidation induced by high-temperature aging drastically reduce the amount of short-range ordered NiCr lattices generated during heating–cooling calibration, since the Cr atoms required for ordered-structure formation were consumed to form the surface oxide layer. Accordingly, the EMF hysteresis of all specimens after aging at 700 °C (Table 10) was markedly lower than those measured before aging (Figure 4) and after aging at lower temperatures (Table 8 and Table 9).

Although oxidation suppresses overall EMF hysteresis, cold-working deformation remains critical as it Cold-working introduces vacancies and dislocations into the alloy, which lower the activation energy for atomic diffusion and accelerate atomic migration. Below 28%, larger deformation accelerates atomic diffusion and produces lower EMF hysteresis. Nevertheless, within the temperature range of 350 °C to 450 °C, the hysteresis loops of thermocouples with 38% deformation was slightly lower than that of samples with 28% deformation. This indicates that the high deformation degree reduced the number of ordered structures that contributed to hysteresis generation. This behavior might arise from the accelerated outward diffusion of Cr atoms under high deformation, which promotes faster and more complete formation of the surface oxide layer. The Cr atoms are more thoroughly depleted by oxidation and are less available for ordered lattice formation. As a result, the correlation between deformation and hysteresis no longer follows a simple monotonic trend; instead, it is modulated by complex metallurgical processes at elevated temperatures.

Under abnormal conditions of a rise in core outlet temperature, for example, during accident working conditions, the hysteresis error of thermocouples with high cold-working deformation may lead to distorted temperature readings and affect judgments of the core safety status. Therefore, the cold-working deformation degree should be strictly controlled during the fabrication of sheathed thermocouple products so as to guarantee comprehensive service reliability under both normal and abnormal reactor operating conditions.

### 3.3. Aging Mechanisms at Different Temperatures

The EMF drift behavior of cold-worked Type-K MIMS thermocouples exhibits pronounced temperature dependence in the range of 350 °C to 700 °C (Figure 5, Figure 6, Figure 7 and Figure 8), indicating that the dominant mechanisms governing EMF variation differ across temperature intervals.

During isothermal aging at 350 °C, the typical core outlet operating temperature of pressurized water reactors (PWRs) (Figure 5), lattice defects (i.e., dislocations and vacancies) introduced by cold-working significantly reduce the activation energy for atomic diffusion, as inferred through classical diffusion theory. At this temperature, short-range ordering (SRO) of Ni and Cr atoms is initiated in the positive KP (Ni–Cr) thermoelement. The slow elemental diffusion, accelerated by lattice defects, combined with the gradual evolution of SRO structures, is proposed as the primary mechanism responsible for the deterioration in EMF stability at 350 °C.

When the aging temperature rises to 500–600 °C, the internal order–disorder structural transformation of the alloy becomes the dominant driver of EMF drift. The data in Table 8 and Table 9 clearly demonstrate that the EMF hysteresis difference during heating–cooling calibration is substantially reduced for all tested samples after aging at 500 °C and 600 °C, confirming that stable, ordered structures have fully formed in the thermoelement alloys after sufficient isothermal treatment. As 600 °C lies near the critical order–disorder transition temperature of the KP alloy, there is still a contribution from ordering but it is partially weakened, leading to slightly higher EMF hysteresis than that observed after aging at 500 °C.

Aging experiments at 700 °C (Figure 8) reveal a distinctly different drift mechanism under high-temperature conditions, where oxidation reactions and rapid outward elemental diffusion take over as the dominant factors for EMF drift. As shown in Table 10, the EMF hysteresis difference increases again after aging at 700 °C. This behavior is attributed to the fast and sustained diffusion of Cr atoms toward the alloy surface that form a dense Cr_2_O_3_ oxide layer. This outward diffusion greatly reduces the Cr atoms available for short-range ordered structure formation, thereby affecting the hysteresis characteristics.

All samples exhibit negative EMF drift at 700 °C and the EMF values of samples with a deformation degree exceeding 28% remain markedly below their initial values after 500 h of aging. This observation directly verifies that high-temperature oxidation and elemental diffusion impose irreversible compositional and microstructural damage in thermocouple alloys, which accounts for the irreversible EMF degradation at high levels of cold-working deformation.

## 4. Conclusions

Considering the requirements for developing combination fixed in-core detector assemblies for pressurized water reactor nuclear power plants, in this study, the influence of cold-working deformation on the electromotive force (EMF) of Type-K sheathed thermocouples was investigated using drawing deformation to simulate the additional deformation stress introduced during thermocouple manufacturing.

Cold-working deformation reduces the calibration EMF of Type-K MIMS thermocouples and increases negative deviation at higher deformation levels, where the drift is dominated by the positive KP thermoelement.

Increased deformation enhances lattice defects and lowers the diffusion activation energy, leading to larger heating–cooling EMF hysteresis and more significant EMF fluctuations during aging at 350 °C. After aging at 500 °C and 600 °C, stable ordered structures form in the thermoelement alloys and hysteresis is substantially reduced. Within the range investigated in this study, deformation degrees exceeding 28% induce irreversible EMF degradation.

To meet nuclear power measurement requirements, cold-working deformation during thermocouple assembly must be strictly controlled to ensure long-term accuracy and stability under both normal and abnormal operating conditions.

## Figures and Tables

**Table 1:** Calibration characteristics of the thermocouple wires.

	Calibration Temperature (°C)							
	100	200	300	400	500	600	800	1000
EMF (μV)	4114	8178	12,168	16,432	20,619	24,989	33,349	41,354

**Table 2:** Nominal compositions of thermocouple wires (Wt.%).

Elements	Thermocouple Alloys	
	KP	KN
Si	0.5	1.5
Cr	10.5	/
Al	/	0.8
Mn	/	2.1
Co	0.7	0.8
Fe	/	/
Ni	Balance	

**Table 3:** Weight percentages of MgO powder and impurities (wt.%).

Element	MgO	CaO	Al_2_O_3_	Fe_2_O_3_	SiO_2_	C	S	B	Cd	B + Cd
Content (%)	99.56	0.072	0.12	0.018	0.040	0.026	0.0035	0.0010	<0.00005	0.0010

**Table 4:** Nominal compositions of 316L stainless steel (Wt.%).

Elements	Si	Cr	Al	Mn	Fe	Mo	Ni
Content (%)	≤1.0	16~18	/	≤2	Balance	2.2	10~14

**Table 5:** Dimensions of the thermocouples.

Sheath Outside Diameter (mm)	Sheath Wall (mm)	Wire Diameter (mm)
3.2 ± 0.03	0.32	0.480
3.0 ± 0.03	0.30	0.450
2.7 ± 0.03	0.27	0.405
2.5 ± 0.03	0.25	0.375

**Table 6:** Calibration characteristics of individual thermoelements relative to platinum at 28% deformation.

EMF (μV)	Calibration Temperature (°C)									
	100	200	300	400	500	600	700	800	900	1000
KP	−27	−65	−79	−115	−122	−124	−122	−128	−130	−132
KN	−9	−15	−19	−23	−26	−30	−30	−23	−22	−28

**Table 7:** EMF variations before and after aging.

Deformation (%)	EMF (μV)					
	500 °C		600 °C		700 °C	
	Before Aging	After Aging	Before Aging	After Aging	Before Aging	After Aging
0	20,565	20,650	24,874	24,904	29,102	29,152
12	20,540	20,602	24,852	24,925	29,089	29,134
28	20,490	20,553	24,798	24,857	29,021	29,078
38	20,449	20,516	24,752	24,818	28,970	29,036

**Table 8:** Heating–cooling calibration EMF deviations of cold-worked thermocouples after aging at 500 °C.

Calibration Temperature (°C)	ΔE = E_Coling_ − E_Heating_ (μV)			
	Deformation			
	38%	28%	12%	0%
300	0	−2	−3	−3
350	−1	−6	−6	−4
400	1	−2	−1	2
450	−1	−3	−3	−2

**Table 9:** Heating–cooling calibration EMF deviations of cold-worked thermocouples after aging at 600 °C.

Calibration Temperature (°C)	ΔE = E_Coling_ − E_Heating_ (μV)			
	Deformation			
	38%	28%	12%	0%
300	−6	−5	−1	−1
350	−15	−15	−10	−11
400	−9	−8	0	−4
450	−6	−5	1	−3

**Table 10:** Heating–cooling calibration EMF deviations of cold-worked thermocouples after aging at 700 °C.

Calibration Temperature (°C)	ΔE = E_Coling_ − E_Heating_ (μV)			
	Deformation			
	38%	28%	12%	0%
300	16	21	21	14
350	8	13	12	9
400	12	15	15	11
450	4	8	7	5

## Data Availability

The data presented in this study are available in the article.
